# Origins of juvenile green sea turtles (*Chelonia mydas*) in the Bahamas: A comparison of recent and historical rookery contributions

**DOI:** 10.1002/ece3.9548

**Published:** 2022-11-27

**Authors:** Camille Kynoch, Mariana M. P. B. Fuentes, Peter H. Dutton, Erin L. LaCasella, Ian Silver‐Gorges

**Affiliations:** ^1^ Department of Earth, Ocean and Atmospheric Science Florida State University Tallahassee Florida USA; ^2^ Marine Mammal and Turtle Division, Southwest Fisheries Science Center, National Marine Fisheries Service National Oceanic and Atmospheric Administration La Jolla California USA

**Keywords:** marine turtles, migratory connectivity, mitochondrial DNA, mixed stock analysis, population structure

## Abstract

Conservation of green sea turtles (*Chelonia mydas*) benefits from knowledge of population connectivity across life stages. Green turtles are managed at the level of genetically discrete rookeries, yet individuals from different rookeries mix at foraging grounds; therefore, rookeries may be impacted by processes at foraging grounds. Bimini, Bahamas, hosts an important foraging assemblage, but rookery contributions to this assemblage have never been resolved. We generated mitochondrial DNA sequences for 96 foraging green turtles from Bimini and used Mixed Stock Analysis to determine rookery contributions to this population using 817 and 490 base pair (bp) rookery baseline data. The MSA conducted with 817 bp data indicated that Quintana Roo, Mexico, and Central Eastern Florida contributed most to the Bimini population. The MSA conducted with 490 bp data indicated that Southwest Cuba and Central Eastern Florida contributed the most to Bimini. The results of the second MSA differ from a previous study undertaken with 490 bp data, conducted in Great Inagua, Bahamas, which suggested that Tortuguero, Costa Rica, contributed the most to that foraging assemblage. Large credible intervals in our results do not permit explicit interpretation of individual rookery contributions, but our results do indicate substantial relative differences in rookery contributions to two Bahamian foraging assemblages which may be driven by oceanic currents, rookery sizes, and possibly juvenile natal homing. Our findings may implicate a shift in contributions to the Bahamas over two decades, highlighting the importance of regularly monitoring rookery contributions and resolving regional recruitment patterns to inform conservation.

## Graphical abstract

We generated mitochondrial DNA sequences for 96 foraging green turtles from Bimini, Bahamas, and used Mixed Stock Analysis to determine rookery contributions to this population. This indicated that Quintana Roo, Mexico, and Central Eastern Florida, USA, contributed most to the Bimini population. This differs from a previous study conducted in Great Inagua, Bahamas, which suggested that Tortuguero, Costa Rica, contributed the most to that foraging assemblage and may be due to oceanic currents, rookery sizes, and possibly juvenile natal homing.

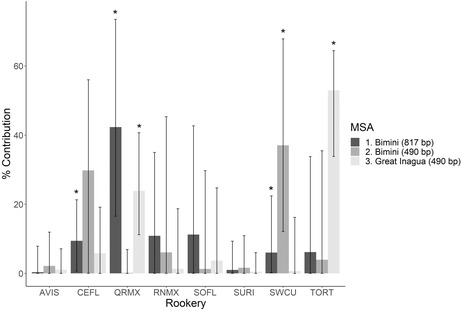

## 1 INTRODUCTION

Understanding genetic population structure is vital to conservation, particularly for highly migratory species that are dependent on multiple, often distant, habitats (Marra et al., 2011; Webster et al., 2002). Knowledge of complex migration and recruitment patterns between natal populations and these habitats requires adequate baseline information on population structure, yet such information is often limited to specific regions (Hulina et al., 2017) or is not frequently updated (National Research Council, 2010). This is further complicated when populations mix throughout their lifecycles (Marra et al., 2011). For instance, minke whales (*Balaenoptera acutorostrata*) make extensive seasonal migrations and conservation efforts have been misdirected due to a lack of information on how breeding populations mix at foraging sites (Anderwald et al., 2011). Bull shark (*Carcharhinus leucas*) conservation is also hindered by a lack of knowledge on the extent and regularity at which individuals from genetically isolated nurseries mix as they migrate between various aquatic habitats (Heupel et al., 2015; Karl et al., 2011). Without an accurate understanding of genetic population structure and connectivity, management actions may fail to reach their goals (Amorocho et al., 2012; Bryan‐Brown et al., 2017; Dubois et al., 2016; Prebble et al., 2018; Worboys et al., 2010).

Green sea turtles (*Chelonia mydas*) often undertake long migrations and frequently mix with individuals from different genetically discrete populations at different life stages (Amorocho et al., 2012; Bowen et al., 1992). Female green turtles exhibit natal homing (Allard et al., 1994; Bowen et al., 1992; Meylan et al., 1990), which results in female turtles engendering genetic population structure among nesting regions with unique sets of mitochondrial DNA (mtDNA) haplotype frequencies (Bowen & Karl, 2007; Shamblin et al., 2016). Sea turtle conservation efforts are generally based on the conservation of turtles originating from these genetically discrete nesting regions, referred to as “management units,” or rookeries, when referring to broader regions (Komoroske et al., 2017; Moritz, 1994). When hatchlings from one rookery leave their natal beach, they spend 1–5 years in the open ocean (Carr, 1987) and eventually migrate to foraging grounds where they frequently mix with individuals from other rookeries as a mixed stock (Bowen & Karl, 2007). Characterizing rookery contributions to foraging assemblages informs effective conservation of green turtle nesting populations, as threats at foraging sites and at nesting beaches may be reflected in the number of turtles a rookery contributes to a foraging assemblage (Hamann et al., 2010; Jensen et al., 2016). For instance, decreased hatchling output from rookeries could eventually cause a quantifiable decline in rookery contributions to foraging assemblages (van der Zee et al., 2019). Quantifying the contributions of rookeries to these mixed foraging sites allows inferences about rookery hatchling production and recruitment (Jensen, 2010). These inferences particularly inform management efforts and give insight into the specific threats faced by turtles from each rookery at their natal beaches and before they recruit to foraging assemblages (Jensen, 2010).

Mixed stock analysis (MSA) is a commonly used technique for determining rookery contributions to mixed stocks (Bolker et al., 2007; Komoroske et al., 2017; Pella & Masuda, 2001). MSA calculates the likelihood that an individual with a certain genetic character in a mixed stock (i.e., a foraging assemblage) originated from a baseline stock (often a genetically discrete rookery, or nesting population within a rookery) with certain genetic character frequencies (Pella & Masuda, 2001). Previous studies have completed MSAs for Caribbean green turtles using mtDNA haplotypes (Bjorndal & Bolten, 2008; Lahanas et al., 1998; Luke et al., 2004; Shamblin et al., 2016). Specifically, studies have focused on the control region of the mitochondrial genome for studying sea turtle population structure and conducting MSAs, as this region is relatively slowly mutating but also contains relatively high nuclear diversity (Avise et al., 1992; Bowen & Karl, 2007). Early studies generated sequences for 490 base pairs (bp) of the control region (Allard et al., 1994; Encalada et al., 1996), which were used in numerous MSAs for sea turtle foraging assemblages (Bass & Witzell, 2000; Bjorndal & Bolten, 2008; Costa Jordao et al., 2017; Lahanas et al., 1998; Luke et al., 2004). Primers were later developed that allowed studies to examine 817 bp of the control region (Abreu‐Grobois et al., 2006). Further, Shamblin et al. (2016) identified a mitochondrial single nucleotide polymorphism (mtSNP) associated with the CM‐A1.1 haplotype in green turtles that provided additionally increased resolution between foraging grounds where CM‐A1.1 is a common haplotype. These advances have allowed for increased discrimination between genetically distinct rookeries and for the quantification of contributions to foraging assemblages with increased accuracy, which in turn provides better insights into the population dynamics and life histories of green sea turtles (Shamblin et al., 2016).

**FIGURE 1:**
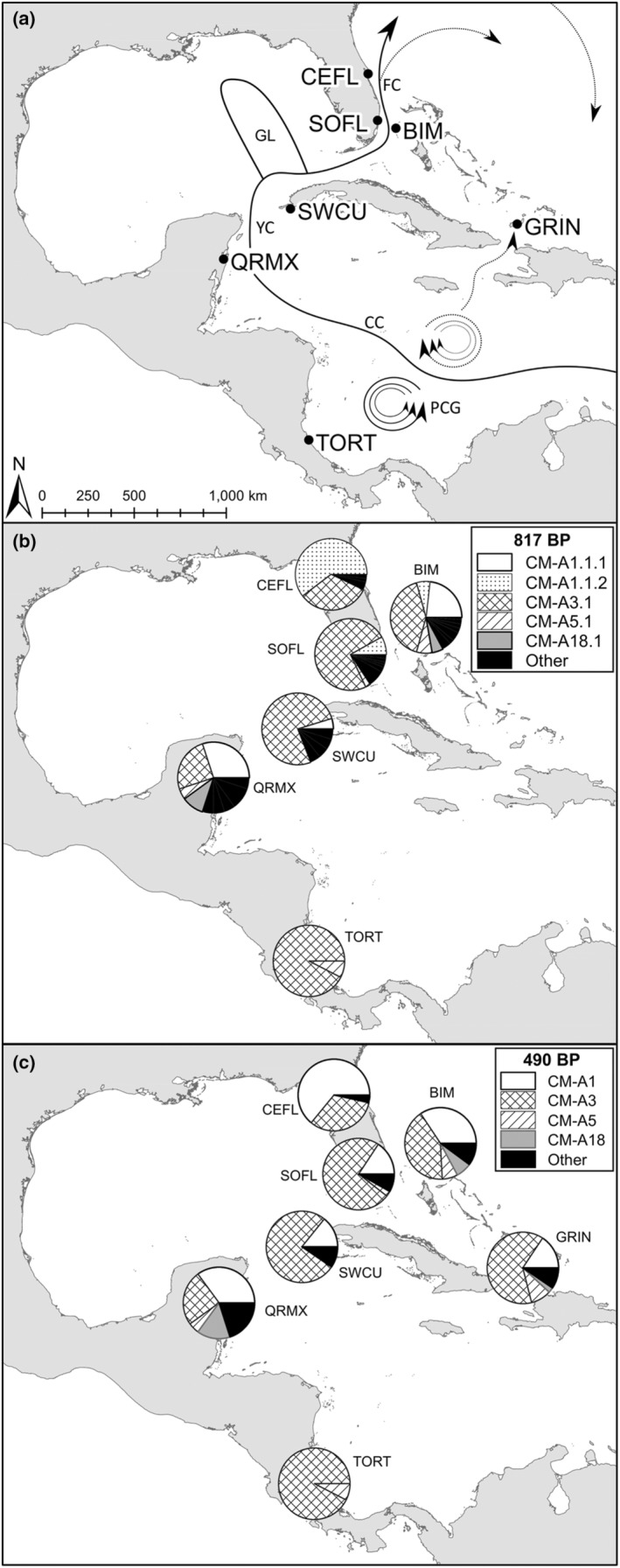
Distribution of haplotypes at major contributing rookeries and study sites, and depiction of currents and small gyres in the surrounding area. Panel a: Sampling sites and approximation of currents in the greater Caribbean that may be influential in juvenile green turtle migration to foraging grounds. Arrows indicate current direction, black lines indicate major current features, and dotted lines indicate possible pathways to the pelagic Atlantic Ocean and Great Inagua. Panel b: 817 bp haplotype proportions at sampling sites where available; panel c: 490 bp haplotype proportions at sampling sites. BIM, Bimini, Bahamas; CC, Caribbean current; CEFL, Central eastern Florida; FC, Florida current; GRIN, Great Inagua, Bahamas; PCG, Panama Colombia gyre; QRMX, Quintana Roo, Mexico; SOFL, South Florida; TORT, Tortuguero, Costa Rica; YC, Yucatan current.

Rookery contributions to the Northwest Atlantic green turtle foraging assemblages are particularly important to quantify because green turtles face threats such as fishing bycatch (Foley et al., 2007; LaCasella et al., 2013), climate change (Komoroske et al., 2017), and habitat degradation (Balazs & Chaloupka, 2004; Hamann et al., 2010) at nesting beaches and foraging grounds and are as such listed as Endangered by the IUCN Red List (Hamann et al., 2010; IUCN, 2021). To date, eight genetically distinct green turtle rookeries have been characterized in the Northwest Atlantic Ocean (NWA; Table S1; Shamblin et al., 2016), in addition to important foraging habitats for green turtles of all life stages (Bjorndal & Bolten, 2008; Gillis et al., 2018; Putillo et al., 2020). The shallow seagrass beds and mangrove estuaries of the Bahamas in particular serve as important foraging areas and refugia for juvenile NWA green turtles from many of these rookeries (Bjorndal & Bolten, 2008; Gillis et al., 2018; Putillo et al., 2020). Early work in this region focused on juvenile green turtles at Great Inagua, Bahamas (Figure 1; Bjorndal & Bolten, 2008; Lahanas et al., 1998), with MSA using 490 bp mtDNA sequence data. This suggested that the Tortuguero, Costa Rica rookery, contributed substantially (79% of individuals) to the juvenile green foraging assemblage at Great Inagua (Lahanas et al., 1998). Later, the relative rookery contributions to the Great Inagua assemblage were reexamined in a longitudinal study, which confirmed the contribution from Costa Rica (Bjorndal & Bolten, 2008). Significant temporal structuring in haplotype frequencies was found among all years, but Costa Rica remained the top contributor with a composite contribution of 58% of individuals over the 12‐year study period (Bjorndal & Bolten, 2008). This structuring was thought to be due to variations in hatchling productivity from contributing rookeries due to considerable regional fluctuations in the number of turtles that nest each year (Bjorndal & Bolten, 2008; Troëng & Rankin, 2005). Alternatively, the variation in hatchling productivity could stem from the long‐term trends in abundance at each rookery (Bjorndal & Bolten, 2008), which might be a signal of the relative efficacy of conservation efforts at these rookeries (Mazaris et al., 2017).

Despite the potential for shifting rookery contributions to Bahamian foraging assemblages over time (Bjorndal & Bolten, 2008; van der Zee et al., 2019), no study to date has confirmed this at other known foraging areas in the Bahamas. The lack of recent genetic analyses of Bahamian turtles means that data used in the previous Bahamian MSAs do not reflect haplotype frequencies representing longer mtDNA sequences and mtSNPs (Shamblin et al., 2016), nor do such haplotype data exist for any Bahamian foraging assemblage. Thus, previous analysis of rookery contributions may not be as accurate as contemporary techniques allow. Additionally, the Bahamas comprise a long chain of islands (>800 km) and it is possible that different Bahamian foraging locations would present with different rookery contributions, perhaps due to the influence of regional ocean currents (Shamblin et al., 2016), rookery sizes (Lahanas et al., 1998), or to juvenile natal homing (Shamblin et al., 2016). Sampling from other known foraging areas (i.e., Gillis et al., 2018; Putillo et al., 2020) might elucidate the differential importance of Bahamian foraging areas to different NWA green turtle rookeries.

Bimini, Bahamas, is the only mangrove and creek habitat on the western edge of the Great Bahamas Bank and it has also been identified as an important foraging site for a resident population of juvenile green turtles (Fuentes et al., 2019; Gillis et al., 2018, 2020; Putillo et al., 2020). Here, we determine rookery contributions to the foraging assemblage in Bimini from 2016 through 2018 using MSA and compare our results to previous MSAs from the Bahamas (Bjorndal & Bolten, 2008). Further, we examine the potential mechanisms driving differences in foraging ground recruitment. Our results provide a basis for future assessments of the impacts of management actions at contributing rookeries on Bahamian foraging assemblages.

## 2 METHODS

### 2.1 Study site and sample collection

Bimini, Bahamas (25° 44′11.36″ N, 79° 16′53.98″ W; Figure 1), is located on the Northwest side of the Great Bahamas Bank, approximately 86 km east of Miami, Florida, U.S.A., and is comprised of two small islands (North and South Bimini) separated by a 0.15‐km wide channel (Putillo et al., 2020). In Bimini, South Bimini and Bonefish Hole have been identified as hotspots for green turtles (Fuentes et al., 2019; Gillis et al., 2018). South Bimini is an open coastal seagrass bed and Bonefish Hole, located to the North, is a mangrove tidal estuary (Gillis et al., 2018). Bimini is <800 km from Great Inagua (Figure 1).

Samples for genetic analysis were obtained across five sampling trips to Bimini consisting of two trips in 2016 (Fuentes et al., 2019; Gillis et al., 2018); one trip in 2017 (Fuentes et al., 2019; Gillis et al., 2020); and two trips in 2018 (Putillo et al., 2020). A total of 135 h of survey effort was completed across the trips to Bimini. Turtles were captured from a moving vessel via the rodeo method (Limpus & Walter, 1980). Once turtles were brought onboard, straight and curved carapace lengths were measured (±0.1 cm; SCL and CCL, respectively), following the protocols described in Pritchard et al. (1999). Body weight (W, ±0.1 kg) was obtained using a hanging balance (Pesola, PHS100). Each turtle was checked for Inconel flipper tags (National Band and Tag Company, Style 681) and a passive integrated transponder (PIT tag, Biomark, GPT12; Pritchard et al., 1999). If none were present, coded Inconel flipper tags were added on the trailing edge of both front flippers and a PIT tag was inserted sub‐dermally in the front left flipper. Epidermis (i.e., skin) samples were collected from the dorsal surface of the neck using a sterile razor blade (Lemons et al., 2011). This technique allowed for collection of epidermis only and no underlying connective tissue (Lemons et al., 2011). Epidermis samples were then placed in a vial with dry salt for preservation and stored at room temperature as per Gillis et al. (2018). Each turtle was marked with All‐Weather PAINTSTIK Livestock Marker in order to avoid recapture.

### 2.2 DNA extraction and sequencing

Skin samples were shipped to the Southwest Fisheries Science Center in La Jolla, California, for laboratory processing and long‐term storage. Genomic DNA was extracted from each sample using a modified sodium chloride (NaCl) procedure (Miller et al., 1988). A fragment (~817 bp) of the mtDNA control region was amplified and sequenced as summarized in LaCasella et al. (2013). Briefly described, polymerase chain reactions (PCR; 25 μl) were conducted using primers LCM15382 (5′ GCT TAA CCC TAA AGC ATT GG 3′) and H950g (5′ GTC TCG GAT TTA GGG GTT TG 3′; Abreu‐Grobois et al., 2006) and standard laboratory methodologies. The PCR cycling profile consisted of initial DNA denaturation at 94°C for 2 min, followed by 30 cycles of (1) denaturation at 94°C for 50 s, (2) annealing at 56°C for 50 s, and (3) extension at 72°C for 1 min, and (4) and a final extension of primers at 72°C for 5 min. Negative controls were included in each PCR to detect contamination and products were verified on 2% agarose gels (Maniatis et al., 1982). PCR products were purified using Exonuclease I and Shrimp Alkaline Phosphate solution (USB) then cycle sequenced with both H950g and LCM15382 using an ABI® Big Dye™ Terminator v3.1 Cycle Sequencing Kit and analyzed with Applied Bio‐systems® (model 3730) automated genetic analyzer. The CmA1.1 haplotypes in our dataset were further evaluated for mtSNP state (Shamblin et al., 2012). Following the same conditions as described for CmA5 in Shamblin et al. (2012), primers 12351F and 13495R (See table S1 in Shamblin et al., 2012) were used for PCR and the samples were sequenced in one direction using primer 12781F.

### 2.3 Data analysis

MtDNA control region sequences were edited and analyzed using the program Geneious v 8.1 (Kearse et al., 2012) and haplotypes were assigned by comparing the sequences to a reference database consisting of published and unpublished Atlantic green turtle haplotypes located on the Archie Carr Center for Sea Turtle Research website (http://accstr.ufl.edu/resources/mtdna‐sequences). The CMA1.1 samples were aligned to each other in Geneious to look at the mitogenomic position 12,958, representing two variants CmA1.1.1 and CmA1.1.2, to further distinguish between northern (Florida) and southern populations (Rancho Nuevo, Mexico). AMOVAs and pairwise F_ST_ were calculated in Arlequin v. 3.5.2.2 between sampling years and foraging sites (South Bimini and Bonefish Hole) to determine how to group the data for the MSA (Excoffier & Lischer, 2010). Haplotype (*h*) and nucleotide (*π*) diversity were calculated using DnaSP V to quantify genetic diversity in Bimini (Rozas et al., 2017).

Three MSAs were conducted to determine rookery contributions to Bahamian green turtle foraging assemblages using Bayesian approaches implemented in BAYES (Pella & Masuda, 2001; Table 1). BAYES, which estimates rookery contributions from multiple potential rookeries to one foraging assemblage (mix) at a time, was chosen over “mixstock” which can include multiple foraging populations in one analysis with a “many‐to‐many” model (Bolker et al., 2007), since the latter is more appropriate when all the many foraging assemblages in a metapopulation have been adequately sampled (Jensen et al., 2016). The first MSA (MSA1) used the highest‐resolution control region (817 bp) and mtSNP data to estimate rookery contributions to Bimini foraging assemblages. The 817 bp and mtSNP Bimini haplotypes found here served as the mixed stock, and 817 bp and mtSNP haplotypes from Shamblin et al. (2016) served as baseline rookery data (Table S1). Individual MCMC chain length was 1666, the burn‐in was half the MCMC chain length (833), and the total MCMC sample for all chains combined was 6664. The second (MSA 2) and third (MSA 3) MSAs allowed for a comparison of rookery contributions to Bimini and Great Inagua (as reported in Bjorndal & Bolten, 2008). MSA 2 used 490 bp haplotype data from Bimini as the mixed stock and 490 bp haplotype data from Shamblin et al. (2016) to estimate rookery contributions to Bimini. Chain convergence was confirmed by checking the estimates of the Gelman and Rubin Diagnostic (as implemented in BAYES) were <1.2 (Pella & Masuda, 2001). Individual MCMC chain length was 4524, the burn‐in was 2262, and the total MCMC sample was 18,096. The Bimini haplotypes in MSA 2 had to be reduced to 490 bp so the results could be accurately compared to the most recent available data from Great Inagua, which are 490 bp haplotype data. MSA 3 used 490 bp haplotype data from Great Inagua (see electronic appendix table A1 from Bjorndal & Bolten, 2008) as the mixed stock and 490 bp haplotype data from Shamblin et al. (2016) to estimate rookery contributions to Great Inagua. Individual MCMC chain length was 6586, the burn‐in was 3293, and the total MCMC sample was 26,344. MSA 3 also investigated if using more recent baseline rookery data provided different rookery contribution estimates to Great Inagua relative to older baseline rookery data used in Bjorndal and Bolten (2008). While some previous studies choose to weight priors based on rookery size (Chaves et al., 2017; Proietti et al., 2012; Shamblin et al., 2016), equal priors were used for all MSAs in this study. NWA green turtle rookeries are sufficiently differentiated by 817 bp and mtSNP data, and MSAs utilizing 490 bp data in this region (e.g., MSAs 2 and 3 here) may be overly sensitive to such weighting (Read et al., 2015; Shamblin et al., 2016, 2018). All MSAs were run with eight independent chains (one per baseline rookery in Shamblin et al., 2016) and equal initial stock contributions among rookeries. MSA results were compared qualitatively to determine differences in rookery contributions between Great Inagua and Bimini and are presented here with 95% credible intervals (95% CI).

**TABLE 1:** Description of MSAs conducted in this study.

	Mixed stock (foraging ground)	Baseline stock (rookery)	Haplotype sequence length	Purpose
MSA 1	Bimini (present study)	Shamblin et al. (2016)	817 bp	To determine rookery contributions to Bimini
MSA 2	Bimini (present study)	Shamblin et al. (2016)	490 bp	To accurately compare rookery contributions from Bimini to Great Inagua, and to show discrepancies in results when using different sequence length data
MSA 3	Great Inagua (Bjorndal & Bolten, 2008)	Shamblin et al. (2016)	490 bp	To accurately compare rookery contributions from Bimini to Great Inagua

## 3 RESULTS

### 3.1 Turtles sampled

A total of 96 turtles were captured in Bimini between 2016 and 2018 (26 at Bonefish Hole and 70 at South Bimini). Turtle sizes ranged from 22.2 to 63.9 cm SCL (Bonefish Hole mean: 39.5 cm ± 6.29 SD; South Bimini mean: 45.68 cm ± 8.28 SD), and 24.5 to 69.9 cm (Bonefish Hole mean: 42.09 cm ± 6.81 SD, South Bimini mean: 48.5 cm ± 9.18 SD). Weights ranged from 1.2 to 35.1 kg (Bonefish Hole mean: 8.39 kg ± 4.54 SD, South Bimini mean: 12.82 kg ± 6.95 SD).

Fourteen haplotypes were found in Bimini between 2016 and 2018, including one orphan haplotype CM‐A2.2 (GenBank ID: MT833385), which is currently unassigned to any rookery (Table S1). The most prominent haplotype in the Bimini assemblage was CM‐A3.1 (42.71%, *n* = 41) followed by CM‐A1.1 (28.13%, *n* = 27, Table S1). All other haplotypes comprised 29.17% of the Bimini foraging assemblages, with little variation in haplotype frequencies between years (as demonstrated by *F*_ST_ and AMOVA results, see below). Total haplotype diversity (*h*) at Bimini was 0.114 and nucleotide diversity (*π*) was 0.00347.

### 3.2 Rookery contributions

**FIGURE 2:**
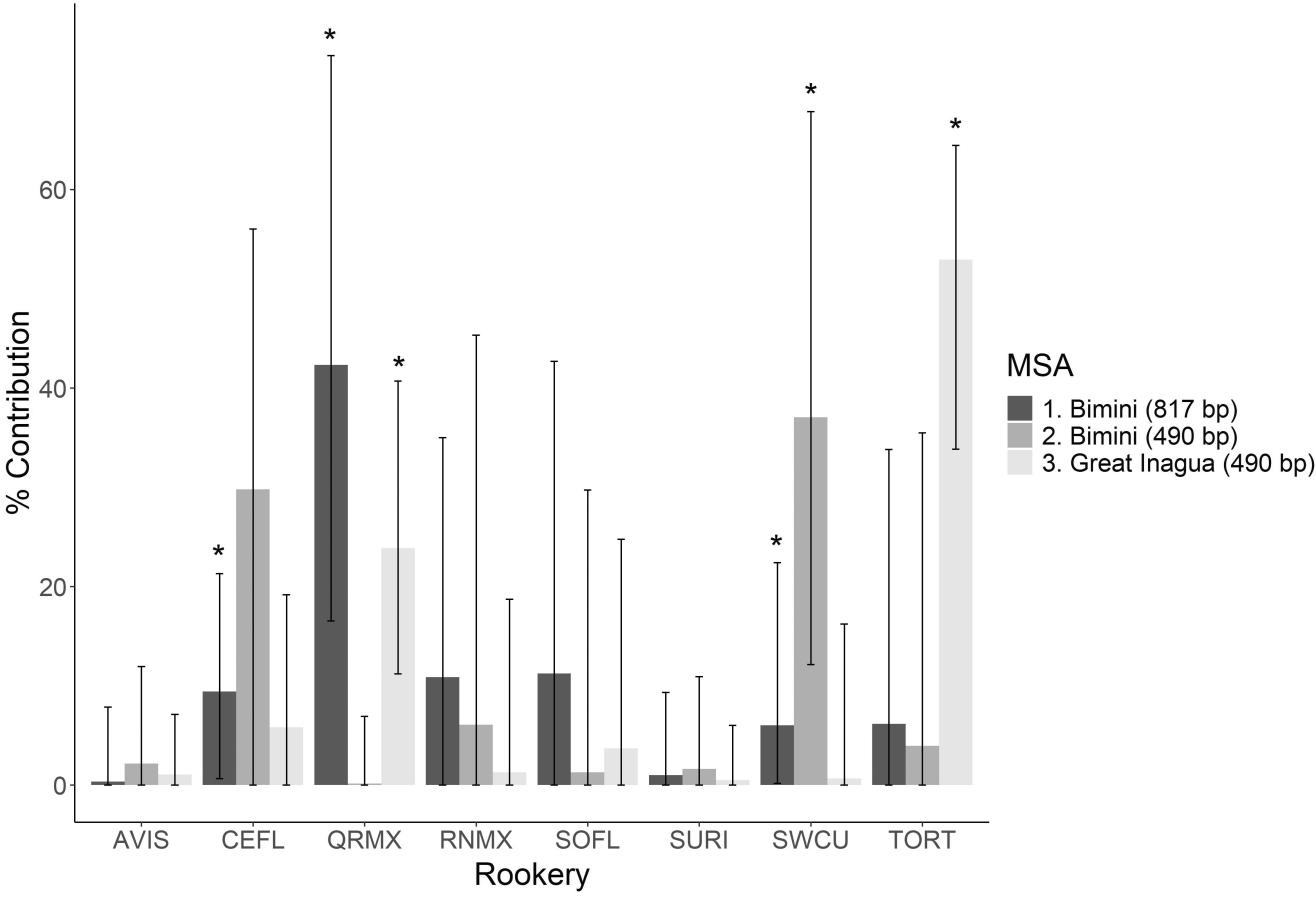
Results from three mixed stock analyses (MSAs) of juvenile green turtle foraging grounds. Dark gray – 817 bp Shamblin et al. (2016) rookeries and Bimini data (MSA 1); medial gray – 490 bp Shamblin et al. (2016) rookeries, 490 bp Bimini foraging population (MSA 2); light gray – 490 bp Shamblin et al. (2016) rookeries, 490 bp Bjorndal and Bolten (2008) foraging population (MSA 3). * indicates rookeries for which credible interval does not overlap 0. AVIS, Aves Island; CEFL, Central Eastern Florida; QRMX, Quintana Roo, Mexico; RNMX, Rancho Nuevo, Mexico; SOFL, South Florida; SURI, Surinam; SWCU, Southwest Cuba; TORT, Tortuguero, Costa Rica.

Pairwise *F*_ST_ and AMOVA did not show significant structuring between years or locations (*p* > .05 in all tests). Therefore, the entire Bimini dataset was treated as one population for all MSAs. MSA 1 indicated that the major contributors to the Bimini foraging ground were Quintana Roo, Mexico (42.3%, 95% CI [17.5%, 73.5%]), Central Eastern Florida (9.4%, 95% CI [0.7%, 21.3%]), and Southwest Cuba (6.0%, 95% CI [0.2%, 22.4%]; Figure 2).

MSA 2 produced different results than MSA 1 (Figure 2) and indicated that Southwest Cuba (37.0%, 95% CI [12.2%, 67.9%]) and Central Eastern Florida (29.8%, 95% CI [0.02%, 56.0%]) contributed the most to the Bimini foraging assemblage. MSA 3 revealed that Tortuguero, Costa Rica, was the main contributor to the foraging assemblage at Great Inagua (53.0%, 95% CI [33.8%, 64.5%]), while Quintana Roo was responsible for 23.9% (95% CI [11.2%, 40.7%]) of turtles at this foraging assemblage. These results agree with the composite results in the previous study at Great Inagua, with approximately 60% of contributions from Tortuguero and approximately 20% from Great Inagua (Bjorndal & Bolten, 2008).

## 4 DISCUSSION

Quintana Roo and Central Eastern Florida, respectively, contributed the most turtles to the foraging assemblage at Bimini according to the highest resolution (817 bp), contemporary baseline rookery data (MSA 1). While lower‐resolution data (490 bp; MSA 2) suggested that Southwest Cuba, and not Quintana Roo, contributed most to the Bimini foraging assemblage, this is likely an artifact of decreased discrimination between rookeries when using lower‐resolution sequence data. The need for contemporary, high‐resolution genetic data at sea turtle foraging grounds globally is highlighted by contrasts between MSAs 1 and 2 conducted for Bimini green turtles. In this case, using lower‐resolution data led to different, likely inaccurate rookery contribution estimates. Rookery contributions to Bimini contrast to rookery contributions to Great Inagua, which hosts turtles primarily from Tortuguero and Quintana Roo, respectively, as originally reported by Bjorndal and Bolten (2008) and confirmed here. We caution against over‐interpretation of the point estimates of individual rookery contributions due to large credible intervals in our results, and because the data from Great Inagua (Bjorndal & Bolten, 2008) are not directly comparable with the data we analyzed here. Nevertheless, our estimates do signal relative differences in rookery contributions that merit attention. The distinction in rookery contributions between these two foraging assemblages could be due to ocean currents, rookery size, juvenile natal homing, or the interplay of these factors (Bass et al., 2006; Bass & Witzell, 2000; Bolker et al., 2007; DuBois et al., 2021; Lahanas et al., 1998; Luke et al., 2004; Naro‐Maciel et al., 2006; Okuyama et al., 2009; Shamblin et al., 2016).

Ocean currents are known to influence juvenile turtle dispersal in this region (Bass et al., 2006; DuBois et al., 2021; Shamblin et al., 2016). Hatchling sea turtles swim toward the nearest offshore currents in part to facilitate dispersal (Salmon & Wyneken, 1987; Wyneken & Salmon, 1992) and immature green turtles ride currents when recruiting to neritic foraging areas before returning to foraging areas closer to their natal beach as adults (DuBois et al., 2021; Okuyama et al., 2009; Witham, 1980). An examination of currents in the Caribbean and Atlantic may explain the genetic composition of foraging turtles at Bimini and Great Inagua (Figure 1). The Caribbean, Yucatan, and Florida Currents, which precede the Gulf Stream, pass offshore of Central America, then run adjacent to Quintana Roo and Cuba, wrap around the Northwest side of Cuba, run between the Florida Keys and Cuba, and then turn north along Florida's east coast, directly adjacent to Bimini (Figure 1; Leaman et al., 1987). The time to recruitment to juvenile foraging grounds for green turtles in this region is thought to be approximately 5 years (Putman & Mansfield, 2015; Reich et al., 2007), and it is likely that post‐hatchling green turtles would ride the Gulf Stream north into the Atlantic Gyre and recruit to Bimini and Great Inagua after developing into juveniles in the pelagic Atlantic Ocean (Carr, 1987).

It is possible that turtles originating from Costa Rica and elsewhere in the main Caribbean basin may become trapped in the circulation of smaller gyres (Figure 1; Luke et al., 2004). Post‐hatchling turtles from Tortuguero, for example, could develop within the Caribbean and be transported toward Jamaica, Hispaniola, and Cuba via these small gyres (Blumenthal et al., 2009), and then subsequently recruit to the Great Inagua foraging assemblage as juveniles (Figure 1; Barbour et al., 2020; Jensen et al., 2016). Juvenile green turtles are known to forage within the Caribbean (Bass et al., 1998; Campbell & Lagueux, 2005), and it is possible that some develop within the Caribbean, feeding within sargassum mats trapped within these gyres (Troeng et al., 2005). This is supported by particle tracking (Blumenthal et al., 2009) and satellite tags (Troeng et al., 2005) from Tortuguero showing entrapment within Caribbean gyres. However, juveniles reported foraging within the Caribbean are typically larger (minimum 67.4 cm SCL; Campbell & Lagueux, 2005) than those reported here from Bimini (maximum 69.9 SCL), and it is more likely that juvenile green turtles recruit into the Caribbean from the Bahamas and Antilles at larger sizes, rather than developing within the Caribbean. Ultimately, there is a need for long‐term tracking of post‐hatchling and juvenile NWA green turtles to resolve dispersal from natal rookeries and subsequent recruitment to foraging assemblages.

Juvenile NWA green turtles may display natal homing when recruiting to Bimini and Great Inagua from the Atlantic Ocean, as is demonstrated by juvenile NWA green turtles from other rookeries (Shamblin et al., 2016) and by juveniles of other sea turtle species and populations (Bass et al., 2006; Bowen et al., 2004; Engstrom et al., 2002; Laurent et al., 1998; Monzón‐Argüello et al., 2010; Naro‐Maciel et al., 2012). Our results might support this for Caribbean Rookeries (Figure 1). Molecular diversity metrics of the foraging assemblage at Bimini were lower than those of other foraging assemblages of northwest Atlantic juvenile green turtles (Great Inagua: *h* = 0.3703, *π* = 0.0064, Bass et al., 2006; Lahanas et al., 1998; Central Eastern Florida: *h* = 0.6015, *π* = 0.0017; Reusche, 2020), and were also lower than those of the Quintana Roo rookery (*h* = 0.8158, *π* = 0.0051; Bass et al., 2006; Encalada et al., 1996), which is Bimini's main contributor as identified here. Molecular diversity metrics at Bimini were most similar to those from Tortuguero (*h* = 0.16, *π* = 0.0034; Bjorndal et al., 2005). Lower molecular diversity at Bimini relative to other foraging grounds in this region may be due to selective recruitment to Bimini by just a portion of turtles originating from Quintana Roo. Great Inagua had substantial contributions from Tortuguero, Quintana Roo, and Central Eastern Florida (Bjorndal & Bolten, 2008), while MSA 1 here suggested that contributions to Bimini from rookeries other than Quintana Roo were minor (<23%, Figure 2). Selective recruitment could be due in part to some aspect of Bimini's geography relative to Quintana Roo (Figure 1). If northwest Atlantic juvenile green turtles display any natal homing when recruiting to foraging assemblages, then individuals that recruit from the Atlantic to Bimini that do not originate from Quintana Roo may move elsewhere, while those from Quintana Roo would concentrate in Bimini, among other foraging grounds (e.g., Great Inagua; Bjorndal & Bolten, 2008). This paradigm would explain why the assemblage at Bimini has lower diversity than other foraging assemblages and even than its main contributing rookery. However, we are unable to make any conclusions regarding natal homing with confidence based on the available data, which come from separate decades and, in the case of Great Inagua, showed inherent variability over time which may be a function of rookery size (see below; Bjorndal & Bolten, 2008). Future studies should aim to comprehensively sample juvenile green turtles at multiple foraging grounds across the NWA, and generate 817 bp and mtSNP data (or use other markers; i.e., genomic SNPs) to determine the extent to which natal homing might influence recruitment in this region.

Rookery size is likely a factor in determining rookery contributions to foraging assemblages, whereby larger rookeries should contribute more turtles to foraging assemblages than smaller rookeries (Lahanas et al., 1998). If rookery size influences rookery contributions to Bahamian foraging grounds, the historical comparison between Bimini and Great Inagua may have implications for abundances at the primary contributing rookeries. The results of MSA 1, that Quintana Roo and Central Eastern Florida contribute most to the Bimini foraging assemblage, should reflect more recent population sizes (and hatchling production) at contributing rookeries than should Bjorndal and Bolten's (2008) MSA for Great Inagua. Further, across the 12 years of data collection at Great Inagua, the percent contribution from Tortuguero decreased, while percent contribution from Florida and Quintana Roo increased (Bjorndal & Bolten, 2008). This trend, and the results of MSA 1, could be due to increasing abundances at the Florida and Quintana Roo rookeries relative to the Tortuguero rookery. While it was difficult to find reliable long‐term nesting data for all rookeries used in the MSA, we can evaluate the management efforts implemented at the major contributors to Bimini, since increased abundance at contributing rookeries is expected with historical and ongoing management efforts (Chaloupka & Limpus, 2001). The Marine Turtle Conservation Act (MTCA) was implemented in 2004 and the Florida Marine Turtle Protection Act (FMTPA) was implemented in 2012 (Florida 2004; United States 2012). The MTCA allocated five million dollars in federal funding to sea turtle management and research in other countries to prohibit illegal trading of sea turtles and their eggs (United States 2012). The FMTPA set new regulations in place to prevent harassing, transfer, sale, and take of sea turtles, their eggs, and their nests and implemented rules to protect nesting habitat (Florida 2004). Florida green turtle nest counts have increased eightfold since 1989 (Index Nesting Beach Survey Totals (1989–2020) | FWC, n.d.); therefore, the success of these management actions could account for the increase in contributions from Florida and Quintana Roo to the Bahamas. Since hatchling production in one nesting season could take 5 –10 years to be reflected in foraging populations (Putman et al., 2020; Reich et al., 2007), increases such as these might also reflect delayed dividends of older conservation strategies, such as the Endangered Species Act (ESA), passed in 1973 (United States 1973) and the mandatory use of Turtle Exclusionary Devices in the shrimping industry, implemented in 1987 (United States 1987). The implementation and enforcement of the ESA in the Hawaiian Archipelago resulted in a long‐term, substantial increase in its green turtle population (Balazs & Chaloupka, 2004; Mazaris et al., 2017), and it is possible a similar phenomenon is happening in the Caribbean and Northwest Atlantic Ocean. In addition, in 1990 the Mexican government banned the harvest of sea turtle eggs and sale of sea turtle products and pledged more support to conservation programs (Aridjis, 1990). Sea turtle population numbers can take time to rebound after conservation efforts are implemented, and it is possible these conservation acts could explain the shift between rookery contributions from 2003 (MSA 3; Great Inagua; Bjorndal & Bolten, 2008) and 2016–2018 (MSA 2; Bimini; present study). Patterns of increased recruitment to foraging grounds following conservation actions have been observed in other highly migratory marine species, such as the Southern Hemisphere humpback whale population (Pallin et al., 2018). This population makes extensive migrations between their breeding grounds at equator and feeding grounds in Antarctic waters (Albertson et al., 2018). Since the International Whaling Commission banned the hunting of this population worldwide, there is evidence of growth in the Antarctic foraging population (Pallin et al., 2018).

Factors such as ocean currents, rookery size, and possibly juvenile natal homing influence rookery contributions to foraging assemblages, as postulated here in examining contributions to Bahamian foraging assemblages. To determine how these factors individually and collectively mediate rookery contributions to each foraging assemblage, we need high‐resolution genetic sampling of foraging turtles, knowledge of hatchling dispersal from natal beaches, information of fine‐scale currents that might facilitate connectivity between rookeries and foraging grounds, and contemporary data on rookery sizes. High‐resolution genetic sampling of foraging turtles in the Bahamas will allow us to assess the influence of natal homing on rookery contributions to foraging assemblages. In the Bahamas, we would expect rookery contributions to shift from primarily Quintana Roo and Florida to primarily Tortuguero as foraging green turtles are sampled across the Bahamian Islands from Bimini to Great Inagua. Currents determine where hatchlings reside prior to recruiting to foraging grounds as juveniles (Naro‐Maciel et al., 2012). Hatchling dispersal can be examined using satellite telemetry (Mansfield et al., 2012, 2017), and/or modeled using Lagrangian particle simulations (Hays et al., 2010; Le Gouvello et al., 2020; Shamblin et al., 2016; Wildermann et al., 2017). Such work, if conducted at the major Atlantic green turtle rookeries that contribute to foraging grounds within the Greater Caribbean, could elucidate which directions post‐hatchling turtles disperse from natal beaches, and if they end up in positions that would place the Bahamas en route toward their natal rookeries.

The differences in rookery contributions between Great Inagua and Bimini have implications for sea turtle conservation in this region and elsewhere. Management agencies for rookeries and foraging areas may use rookery contribution data to inform management strategies, by drawing inferences between rookery contributions and vital rates that influence rookery demographics (i.e., recruitment), and factors that subsequently influence those vital rates (such as predation, disease, and anthropogenic mortality; van der Zee et al., 2019). Understanding the controlling factors of population abundance is indispensable in conservation, particularly for highly migratory species such as green turtles (Webster et al., 2002). If rookery contributions deviate from what one expects based on rookery size data and knowledge of hatchling dispersal and currents, this could indicate that there is an unknown source of turtle mortality at the nesting or foraging population or in between the two. If rookery contributions are variable over time (as in Bjorndal & Bolten, 2008 and implicit here), this could be a result of fluctuations in abundance at nesting populations due to changes in management actions, or perhaps other demographic, environmental, or ecological factors. If a species crosses international borders at different stages in their life cycle, it is crucial that management agencies cooperate for more effective and sustainable conservation (Marra et al., 2011). As such, a detailed understanding of the migratory pathways and factors that link rookeries and individual foraging assemblages is critical toward informing effective management strategies.

## AUTHOR CONTRIBUTIONS

**Camille Kynoch:** Conceptualization (equal); formal analysis (lead); investigation (lead); methodology (lead); visualization (equal); writing – original draft (lead); writing – review and editing (lead). **Mariana Fuentes:** Conceptualization (equal); data curation (equal); formal analysis (supporting); funding acquisition (lead); investigation (equal); methodology (equal); project administration (lead); resources (lead); supervision (equal); writing – original draft (supporting); writing – review and editing (equal). **Peter Dutton:** Conceptualization (equal); data curation (equal); formal analysis (supporting); investigation (supporting); methodology (supporting); project administration (supporting); supervision (equal); validation (equal); writing – original draft (supporting); writing – review and editing (supporting). **Erin LaCasella:** Conceptualization (equal); data curation (equal); formal analysis (supporting); investigation (supporting); methodology (supporting); software (equal); supervision (supporting); validation (supporting); writing – original draft (supporting); writing – review and editing (supporting). **Ian M Silver‐Gorges:** Conceptualization (equal); formal analysis (equal); investigation (equal); methodology (equal); supervision (lead); visualization (equal); writing – original draft (equal); writing – review and editing (equal).

## Supporting information


Table S1.

## Data Availability

All mtDNA sequences generated in this paper are already available on the NCBI GenBank database.
