# Supplementary material for: Origins of juvenile green sea turtles (Chelonia mydas) in the Bahamas: A comparison of recent and historical rookery contributions
Source: Ecol Evol. 2022 Nov 27;12(11):e9548. doi: 10.1002/ece3.9548 (PMC9702569; doi:10.1002/ece3.9548)
Supplement: Supplementary file 1 — Table S1. [file ECE3-12-e9548-s001.docx]

Table S1. Haplotype frequencies for rookeries (Shamblin et al. 2016) and foraging assemblages (Bjorndal & Bolten 2008, present study); 1. Estimated rookery sizes from Supplementary Table 2 in Shamblin et al. (2016).

|  | Tortuguero, Costa Rica | Rancho Nuevo, Mexico | Quintana Roo, Mexico | Cuba | SW Florida, United States | CE Florida, United States | Aves Island, Venezuela | Suriname | Bimini, Bahamas  (present study) | Great Inagua, Bahamas (Bjorndal and Bolten, 2008) |
| --- | --- | --- | --- | --- | --- | --- | --- | --- | --- | --- |
| Estimated Rookery Sizes (number of nesting turtles)^1^ | 131,751 | 715 | 18,257 | 363 | 3,286 | 4,931 | 2,833 | 9,406 | - | - |
| CM-A1 | - | *24* | *7* | *3* | *27* | *335* |  |  | *32* | *53* |
| CM-A1.1.1 | - | *24* | *6* | *1* |  |  |  |  | *22* |  |
| CM-A1.1.2 | *-* |  |  |  | *14* | *315* |  |  | *5* |  |
| CM-A1.2 | *-* |  | *1* | *1* | *13* | *19* |  |  | *4* |  |
| CM-A1.4 | *-* |  |  | *1* |  | *1* |  |  | *1* |  |
| CM-A2 | *-* |  |  |  | *4* | *8* |  |  |  | *1* |
| CM-A2.2 | *-* |  |  |  |  |  |  |  | *1* |  |
| CM-A3 | *395* | *7* | *5* | *16* | *127* | *170* | *5* | *1* | *41* | *214* |
| CM-A4 | *1* |  |  |  |  |  |  |  |  |  |
| CM-A5 | *32* |  | *1* |  | *4* | *2* | *62* | *55* | *7* | *31* |
| CM-A5.1 | *32* |  | *1* |  | *4* | *2* | *48* | *55* | *7* |  |
| CM-A5.2 |  |  |  |  |  |  | *14* |  |  |  |
| CM-A6 |  |  |  |  |  |  |  | *2* |  |  |
| CM-A8 |  |  |  |  |  | *1* |  |  | *1* | *7* |
| CM-A10 |  |  |  |  |  |  |  |  |  | *1* |
| CM-A13 |  |  |  |  | *2* | *10* |  |  |  | *1* |
| CM-A14 |  |  |  |  |  |  |  |  |  | *1* |
| CM-A15 |  |  | *1* |  |  |  |  |  |  |  |
| CM-A16 |  |  | *1* |  | *1* | *3* |  |  | *3* | *7* |
| CM-A17 |  |  | *2* |  | *2* |  |  |  | *1* | *5* |
| CM-A18 |  |  | *3* |  | *1* | *1* |  |  | *6* | *8* |
| CM-A18.1 |  |  | *2* |  |  |  |  |  | *5* |  |
| CM-A18.2 |  |  | *1* |  | *1* | *1* |  |  | *1* |  |
| CM-A20 | *2* |  |  |  |  |  |  |  |  | *1* |
| CM-A21 | *3* |  |  |  |  |  |  |  |  | *3* |
| CM-A22 |  |  |  |  |  |  |  |  | *1* | *3* |
| CM-A26 |  |  |  |  |  |  |  |  | *2* | *5* |
| CM-A27 |  |  |  | *1* |  |  |  |  | *1* | *6* |
| CM-A28 |  |  |  | *1* | *3* | *3* |  |  |  | *1* |
| CM-A34 |  |  |  |  |  |  |  |  |  | *1* |
| CM-A47 |  |  |  |  |  |  |  |  |  | *1* |
| CM-A48 |  |  |  | *5* |  |  |  |  |  |  |
| CM-A53 |  |  |  |  | *3* |  |  |  |  |  |
| CM-A56 |  |  |  | *1* |  |  |  |  |  |  |
| CM-A57 |  |  |  | *1* |  |  |  |  |  |  |
